# Development and Validation of a Patient Classification System for Rehabilitation Hospitals Based on Patient and Stakeholders Engagement: A Study Protocol

**DOI:** 10.1111/hex.70505

**Published:** 2025-11-18

**Authors:** Shima Shirozhan, Kian Norouzi Tabrizi, Mahdieh Motie, Rouhollah Mirshafiee, Hasan Mohammadi, Amin Ajalli

**Affiliations:** ^1^ Department of Nursing, Pediatric Neurorehabilitation Research Center University of Social Welfare and Rehabilitation Sciences Tehran Iran; ^2^ Department of Nursing, Iranian Research Center on Aging University of Social Welfare and Rehabilitation Sciences Tehran Iran; ^3^ Department of Nursing University of Social Welfare and Rehabilitation Sciences Tehran Iran; ^4^ Rofaydeh Rehabilitation Hospital Tehran Iran; ^5^ Chief Executive Officer of Rofaydeh Rehabilitation Hospital Tehran Iran; ^6^ Director of Nursing Services University of Social Welfare and Rehabilitation Sciences Tehran Iran

**Keywords:** patient and public involvement, patient classification, people with disability, rehabilitation, stakeholders engagement

## Abstract

**Introduction:**

Patient Classification Systems (PCS) are essential for managing resources and staffing in hospitals. However, existing systems are predominantly nursing‐centric and fail to capture the interdisciplinary nature and complexity of care in rehabilitation settings, leading to inaccuracies in workload assessment and resource allocation. This study aims to develop and validate a novel, context‐specific PCS for rehabilitation hospitals through a participatory approach that actively engages patients and stakeholders.

**Methods:**

This mixed‐methods study protocol comprises three sequential stages. First, a systematic scoping review following Arksey and O'Malley's framework will identify key PCS components from existing evidence. Second, structured expert panel sessions employing a modified Delphi technique will develop a consensus‐based preliminary framework. Third, the developed PCS will undergo rigorous validation through pilot implementation, assessing inter‐rater reliability using Cohen's Kappa and criterion validity against established tools, including the Functional Independence Measure (FIM) and Barthel Index, with explicit sample size calculation and reliability criteria.

**Conclusion:**

The study will generate a validated PCS specifically designed for rehabilitation hospital settings through systematic integration of evidence and stakeholder insights. The resulting tool is expected to enhance workload measurement accuracy, promote equitable resource distribution, and ultimately improve both care quality and operational efficiency in rehabilitation contexts.

**Patient or Public Contribution:**

Stakeholders, including patients, their families, administrators, and multidisciplinary clinical staff, have been engaged from the initial conception of the study. They contributed to defining the research objectives, formulating the scoping review questions, and are integral participants in the co‐design and validation stages, ensuring the resulting system is relevant and applicable to real‐world practice.

**Abbreviations**
FIM: Functional Independence Measure
GRIPP2: Guidance for Reporting Involvement of Patients and the Public
ICC: Intraclass Correlation Coefficient
κ: Kappa coefficient
PCS: Patient Classification System
PRISMA‐P: Preferred Reporting Items for Systematic reviews and Meta‐Analyses extension for Protocols
PRISMA‐ScR: Preferred Reporting Items for Systematic Reviews and Meta‐Analyses Extension for Scoping Reviews
SPIRIT: Standard Protocol Items: Recommendations for Interventional Trials

## 1 Introduction

Healthcare systems constitute a substantial financial burden for governments worldwide, with hospitals representing major cost centres within these systems. Consequently, the effective management of financial and human resources in hospital settings is critically important [1, 2]. Patient Classification Systems (PCS) serve as fundamental tools for evaluating service levels and guiding the strategic allocation of resources. By categorizing inpatients according to care type and intensity, PCS facilitates care delivery assessment, cost estimation for budgeting, clinical workforce planning, and patient outcome evaluation [3, 4, 5].

Internationally, several PCS frameworks—including the Oulu Patient Classification (OPC), RAFAELA, and Zebra systems—have been developed to aid in budgetary control and staffing decisions by defining care domains and average service times. While pioneering in their era, these systems demonstrate limitations that diminish their validity and utility in contemporary rehabilitation contexts [4, 5, 6, 7].

A primary limitation of existing systems is their predominantly nursing‐centric focus, which fails to incorporate essential contributions from rehabilitation disciplines such as physiotherapy and occupational therapy, alongside critical rehabilitation services, including interdisciplinary team meetings. This represents a significant shortcoming given rehabilitation's inherently interdisciplinary nature, where vital services like collaborative care planning are delivered through team‐based approaches [8, 9, 10, 11]. Furthermore, current systems inadequately capture the unique and complex needs of individuals with disabilities, as they do not properly account for therapeutic intervention intensity, disability‐specific risks (e.g., pressure ulcers, falls, aspiration), or the substantial time dedicated to indirect care activities such as patient coordination and family education [8, 9, 10, 11, 12].

Consequently, these classification systems cannot reflect the true complexity of rehabilitation care, leading to the systematic omission of time‐intensive yet crucial services. This inadequacy results in frequent patient misclassification, generating inaccurate workload assessments, inefficient resource allocation, impaired operational planning, and inequitable staff allocation [9, 13, 14].

To address these limitations in current Patient Classification Systems, this study aims to develop and validate a novel PCS specifically designed for rehabilitation hospital settings. Recognizing that accuracy and practical utility of such systems fundamentally depend on clinical relevance, our research employs a participatory co‐design methodology grounded in frontline clinical experience. The study will actively engage key stakeholders—including patients, their families, and multidisciplinary rehabilitation team members—throughout all development stages, following established Patient and Stakeholder Engagement (PSE) principles [15, 16]. We anticipate that this participatory approach will significantly enhance both accuracy and practical applicability, ultimately yielding a tool that is scientifically valid while remaining aligned with real‐world rehabilitation needs. This study protocol represents the initial output of the research project, detailing the systematic development of a PCS for inpatient rehabilitation hospitals using a participatory approach.

## 2 Method

This protocol describes a mixed‐methods study employing a sequential explanatory design, comprising three distinct stages: (1) a systematic scoping review to map existing evidence and identify key components of Patient Classification Systems (PCS); (2) structured expert panel sessions to develop a consensus‐based preliminary PCS framework; and (3) a validation stage employing quantitative methods to assess the reliability and validity of the developed PCS in rehabilitation settings. The study protocol has been developed in accordance with the SPIRIT (Standard Protocol Items: Recommendations for Interventional Trials) guidelines.

Patient and stakeholder engagement is embedded throughout the research process to enhance the relevance, applicability, and quality of the resulting classification system. The initial impetus for developing this system originated directly from stakeholders at the Rehabilitation Hospital, including administrators and clinical staff, who identified critical operational challenges with existing tools. Consequently, the study's overarching objectives and specific research questions were formulated collaboratively to ensure the research addresses real‐world needs and expected outcomes.

The protocol systematically integrates stakeholder input at key stages. Following data extraction in the first research stage, findings will be presented to stakeholders to facilitate their comprehension and prepare for the Expert Panel sessions. Patients and stakeholders will participate in all scheduled meetings, providing insights on several critical aspects. These include identifying primary nursing care activities, determining the time allocation and relative importance of these care activities, and offering perspectives on developing a user‐friendly and practically implementable classification system. Stakeholders have specifically defined their participatory roles, with their involvement in the Expert Panel constituting a central component of the co‐design approach. To ensure methodological rigour, the research team adheres to the GRIPP2 (Guidance for Reporting Involvement of Patients and the Public) framework for planning and reporting stakeholder involvement [17].

### 2.1 First Stage: Systematic Scoping Review

This systematic scoping review will be conducted following the established methodological framework developed by Arksey and O'Malley. The framework will be implemented through five core stages, while the optional consultation stage will not be incorporated in this investigation. The development of this review protocol has been informed by the Preferred Reporting Items for Systematic reviews and Meta‐Analyses extension for Protocols (PRISMA‐P) guidelines, and the reporting of scoping review outcomes will adhere to the PRISMA extension for Scoping Reviews (PRISMA‐ScR) checklist [18, 19, 20, 21].

The research questions for this scoping review were developed through collaborative engagement with key stakeholders at the participating Rehabilitation Hospital. Hospital administrators held dedicated meetings with the research team to present specific concerns and operational challenges, which were subsequently synthesized into five primary research questions: determining the most precise structure for a classification system; establishing the optimal number of levels for a Patient Classification System; identifying essential characteristics of a PCS for inpatient rehabilitation hospitals; specifying items for inclusion in a rehabilitation hospital classification system; and determining the appropriate frequency for classification performance.

The search strategy has been designed to comprehensively capture relevant literature pertaining to Patient Classification Systems in healthcare settings. Key search terms and their synonyms were identified through an initial review of seminal papers and examination of standardized vocabularies, including Medical Subject Headings in PubMed and Emtree in Embase. Core concepts encompass ‘patient classification’, ‘persons with disabilities’, ‘rehabilitation‘, and ‘nursing intensity’. Multiple international databases will be searched from their inception to ensure temporal comprehensiveness, including PubMed/MEDLINE, Scopus, Web of Science Core Collection, Embase, and the Cumulative Index to Nursing and Allied Health Literature. The search strategy will be adapted appropriately for each database's syntax and subject headings. For instance, the PubMed search strategy will combine terms for patient classification with rehabilitation and disability‐related terms. To minimize publication bias and identify additional grey literature, supplementary methods will include reference list scanning, hand‐searching key journals, and targeted Google Scholar searches. All retrieved records will be managed using EndNote X09 citation management software, with duplicate removal performed both electronically and manually before screening.

The study selection process will employ a two‐phase screening approach conducted independently by two reviewers to minimize selection bias. Initially, titles and abstracts will be screened against predefined eligibility criteria, followed by full‐text review of potentially relevant articles. The eligibility criteria are structured around the PCC framework, focusing on systems that classify adult inpatients based on care needs. The Population of interest includes patients in hospital settings with emphasis on rehabilitation, while the Concept encompasses Patient Classification Systems, nursing workload measurement tools, and acuity systems designed to categorize patients based on care needs, dependency levels, or resource utilization. The Context is limited to inpatient rehabilitation hospitals or units. Eligible study types include quantitative, qualitative, and mixed‐methods studies, systematic reviews, and key methodological papers describing PCS development, validation, or components. Grey literature such as official reports and theses will also be considered, while studies with unavailable full texts will be excluded. A pilot calibration exercise will be performed on a random sample of 50 records to ensure consistency, with disagreements resolved through discussion or third‐reviewer consultation. The entire selection process will be documented using a PRISMA flow diagram.

The data charting process will be explicitly guided by the primary research questions to ensure collected data directly addresses study objectives. An iterative approach will be used to develop the data charting table, with stakeholder consultations informing the structure and presentation to enhance practical utility and clarity. Two independent researchers will pilot the draft charting table on a sample of included studies to refine categories, with the final table capturing core information essential for answering the research questions. Any discrepancies in data extraction will be resolved through consensus between researchers.

Results collation and summarization will prioritize formats and channels most accessible and actionable for stakeholders, particularly clinical staff and administrators at the rehabilitation hospital. Their direct input will shape the dissemination strategy, ensuring evidence translation into practical insights that directly inform the subsequent stage of stakeholder engagement in classification framework development. The primary output will be a tailored evidence briefing specifically designed to serve as foundational discussion material for the structured expert panel sessions in stage 2.

### 2.2 Second Stage: Expert Panel

The objective of the expert panel is to develop a consensus‐based preliminary framework for the novel Patient Classification System (PCS) by integrating evidence from first stage with the practical expertise of key stakeholders. To achieve this objective, the scoping review findings will be systematically translated into structured discussion materials. These materials will present four key components for panel consideration: a preliminary structural framework outlining potential classification approaches, a comprehensive inventory of candidate classification items with supporting evidence and importance rankings, proposed care levels with defining characteristics, and evidence‐based recommendations for assessment protocols and reassessment intervals. Through a series of iterative discussions, these components will undergo systematic evaluation, refinement, and consolidation to establish consensus on the final PCS structure, operational definitions, and implementation parameters.

A purposive sampling approach will be employed to recruit a multidisciplinary panel of 12‐15 experts, a sample size consistent with methodological recommendations for expert panel studies to ensure diverse representation while maintaining productive discussion. The panel composition will include clinical and managerial nurses with substantial rehabilitation experience, core rehabilitation team members spanning multiple disciplines, including physiatrists, physiotherapists, occupational therapists, speech therapists, and clinical psychologists, along with hospital administrators and unit managers responsible for resource allocation and staffing decisions. Prospective panel members must meet key inclusion criteria requiring a minimum of 5 years of relevant experience in rehabilitation hospital settings and commitment to participate in all scheduled sessions. To ensure the panel's relevance and credibility, stakeholder representatives from hospital management will be consulted during the recruitment process to nominate and endorse potential experts [22].

The panel process will comprise three structured sessions, each lasting approximately 90‐120 min, conducted in a dedicated meeting room at the Rehabilitation Hospital to facilitate access and contextual relevance. Session scheduling will be determined collectively based on participant availability to maximize attendance. Each session's content and objectives will build sequentially upon both the Stage 1 findings and conclusions from previous meetings, ensuring progressive development of the classification framework.

A modified Delphi technique will guide the consensus‐building process, with sessions facilitated by two members of the research team. Discussions will be structured using a semi‐structured question guide derived from Stage 1 findings. Following discussions on key points, panelists will anonymously rate their agreement levels using real‐time polling software with a 5‐point Likert scale. A pre‐defined threshold of 75% agreement will indicate consensus, while items failing to reach this threshold will be revisited for further discussion in subsequent sessions.

The analysis will integrate both quantitative and qualitative data. Quantitative data from agreement ratings will be analysed using descriptive statistics to document consensus levels for each decision. Qualitative data from audio‐recorded discussions will be transcribed verbatim and subjected to conventional content analysis. This dual approach will capture not only the consensus metrics but also the rationale behind decisions, contextual considerations, and potential implementation barriers, providing comprehensive data to support the development of a robust and practical PCS framework.

The primary objective of this stage is to leverage the evidence synthesized in Stage 1 and the practical expertise of key stakeholders to develop a consensus‐based, preliminary framework for the novel Patient Classification System (PCS). To achieve this, the scoping review findings will be translated into concrete discussion materials for the expert panels. These will include: (1) a preliminary structural framework outlining potential classification models (e.g., categorical typologies vs. weighted scoring systems), (2) a comprehensive list of candidate classification items with their supporting evidence and relative importance rankings from the literature, (3) proposed care levels and their defining characteristics, and (4) evidence‐based recommendations for prerequisite assessment conditions and reassessment intervals. Through iterative panel discussions, these components will be systematically evaluated, refined, and consolidated to establish consensus on the final PCS structure, operational definitions, and implementation parameters.

### 2.3 Third Stage: Validation

The third stage of the study will focus on validating the preliminary PCS framework through a rigorous pilot implementation in clinical wards. This validation will quantitatively assess both the reliability and validity of the developed system, ensuring its robustness for clinical application. A minimum of 100 patient assessments will be included in the validation sample, as determined by a precision‐based calculation for Cohen's Kappa. This sample size ensures sufficient statistical power to detect a Kappa coefficient of 0.8 with a margin of error of 0.1 at a 95% confidence level.

The validation procedure will be conducted by two clinical nurses who will receive comprehensive training on the application of the PCS, including detailed operational definitions, scoring criteria, and clinical examples. Training will continue through calibration exercises until consistent application of the system is achieved across both raters.

Reliability assessment will encompass two key dimensions. Inter‐rater reliability will be evaluated by having both trained nurses independently classify the same patients concurrently during morning rounds. The level of agreement between their simultaneous assessments will be measured using Cohen's Kappa coefficient (*κ*). Additionally, test‐retest reliability will be examined by having the same nurses reclassify a subset of approximately 20 patients after a 24‐h interval, assuming no major clinical changes have occurred. The temporal stability of classifications will be assessed using the Intraclass Correlation Coefficient (ICC).

Criterion validity will be established by comparing PCS scores with concurrently administered, established patient dependency measures. The system's outcomes will be correlated with scores from the Functional Independence Measure (FIM), the Barthel Index, and recorded Nursing Care Hours per Patient Day using Pearson's correlation coefficients.

For interpretation, inter‐rater and test‐retest reliability coefficients (Kappa/ICC) exceeding 0.8 will be considered excellent, values between 0.6 and 0.8 will be classified as good, and values below 0.6 will indicate poor agreement. Criterion validity will be demonstrated by moderate to strong positive correlations (*r* > 0.5) with the established measures. All statistical analyses will be performed using SPSS version 26, with descriptive statistics summarizing patient characteristics and reliability/validity coefficients reported with 95% confidence intervals.

The comprehensive findings from this validation study will identify necessary refinements to ensure the final PCS demonstrates adequate psychometric properties for reliable and valid implementation in rehabilitation settings, ultimately contributing to accurate workload assessment and resource allocation.

### 2.4 Ethical Consideration

This protocol is part of a comprehensive research project to be conducted at Rofaydeh Rehabilitation Hospital. The study proposal has undergone scientific and ethical review and is in the process of obtaining ethical approval from the University of Social Welfare and Rehabilitation Sciences Ethics Committee. The research will adhere to all ethical principles outlined in the Declaration of Helsinki, including voluntary participation, written informed consent, confidentiality of data, and the right to withdraw at any time without consequences.

Participation in specific data collection stages ‐ including the expert panel (Stage 2) and validation study (Stage 3)—is strictly voluntary. All participants will receive comprehensive information about the study's purpose and procedures, and written informed consent will be obtained before enrolment. Patient and Stakeholder Engagement, involving patients, families, and healthcare staff, will be maintained throughout all research stages with transparent communication of study objectives and clear definition of roles and expectations. All stakeholder contributions will be systematically documented and appropriately acknowledged in research outputs [16, 22].

## 3 Conclusion

This study presents a structured protocol for developing and validating a Patient Classification System (PCS) tailored to the interdisciplinary nature of rehabilitation hospitals. By integrating evidence from a systematic scoping review, expert consensus, and quantitative validation, the proposed PCS addresses key limitations of existing nursing‐centric models and reflects the complexity of rehabilitation care. The participatory design—engaging patients, families, and multidisciplinary clinical staff—ensures that the system is both scientifically robust and practically applicable. The validated PCS is expected to improve workload measurement accuracy, support equitable resource allocation, and enhance care quality and operational efficiency in rehabilitation settings. Future implementation of this system may contribute to more transparent staffing decisions, better alignment of care intensity with patient needs, and improved interdisciplinary coordination across rehabilitation services.

## Author Contributions

**Shima Shirozhan:** conceptualization, methodology, investigation, project administration, writing – original draft, validation, supervision. **Kian Norouzi Tabrizi:** conceptualization, methodology, investigation, writing – review and editing, design, and framework development. **Mahdieh Motie:** investigation, visualization, writing – original draft, writing – review and editing, design, and framework development. **Rouhollah Mirshafiee:** writing–review and editing, design and framework development, resources. **Hasan Mohammadi:** writing – review and editing, design and framework development, resources, funding acquisition. **Amin Ajalli:** writing – review and editing, design and framework development, resources.

## Ethics Statement

This study has been submitted to be reviewed by the ethics committee of the University of Social Welfare and Rehabilitation Sciences, Tehran, Iran, to obtain an ethics approval code.

## Consent

The authors have nothing to report.

## Conflicts of Interest

The authors declare no conflicts of interest.

## Data Availability

The data sets used and/or analysed during the current study are available from the corresponding author upon reasonable request.
